# Digitale Hochschullehre im ersten COVID-19-Semester. Ergebnisse einer Befragung von Lehrenden in Public Health, Medizin und Pflege

**DOI:** 10.1007/s11553-022-00937-1

**Published:** 2022-03-20

**Authors:** Maria A. Marchwacka, Joachim Kugler, Tom Schaal, Daniel Tolks

**Affiliations:** 1 Lehrstuhl Gesundheits- und Pflegedidaktik, Fakultät für Pflegewissenschaft, Vinzenz Pallotti University, Vallendar, Deutschland; 2 Lehrstuhl Gesundheitswissenschaften/Public Health, Institut für Arbeits- und Sozialmedizin, Medizinische Fakultät, TU Dresden, Dresden, Deutschland; 3 Professur für Management im Gesundheitswesen, Fakultät Gesundheits- und Pflegewissenschaften, Westsächsische Hochschule Zwickau, Zwickau, Deutschland; 4 Zentrum für angewandte Gesundheitswissenschaften, Leuphana Universität Lüneburg, Lüneburg, Deutschland; 5 AG Digitale Medizin, Medizinische Fakultät Bielefeld, Bielefeld, Deutschland

**Keywords:** Hochschuldidaktik, Digitale Transformation, Kompetenzerwerb, Digitale Lernarrangements, Digitale Infrastruktur, Higher education didactics, Digital transformation, Competence achievement, Technological resources, Digital learning arrangements

## Abstract

**Hintergrund:**

Die COVID-19(„coronavirus disease 2019“)-Pandemie hat die Ad-hoc-Digitalisierung an Hochschulen vorangetrieben. Zugleich stand die digitale Hochschullehre vor der Herausforderung der Ausgestaltung der Lehre im Zusammenhang mit den vorbestehenden Ressourcen, der digitalen und didaktischen Kompetenzen sowie der zur Verfügung stehenden technischen Infrastruktur.

**Ziel der Arbeit/Fragestellung:**

Das Ziel der Umfrage war die Einschätzung der digitalen Lehre, die die Präsenzlehre infolge der COVID-19-Pandemie zum großen Teil bzw. gänzlich ersetzt hat, aus der Perspektive der Lehrenden in Public Health, Medizin und Pflege.

**Material und Methode:**

Die Querschnitterhebung fand online von Juni bis August 2020 statt und die Daten wurden über www.soscisurvey.de erhoben. Die schriftliche Befragung wurde unter Mitgliedern der Deutschen Gesellschaft für Public Health (DGPH) und des Ausschusses Digitalisierung der Gesellschaft für medizinische Ausbildung (GMA) und der Sektionen Bildung und Beratung der Deutschen Gesellschaft für Pflegewissenschaft (DGP) sowie der Arbeitsgruppe Lehre der Deutschen Gesellschaft für Medizinische Soziologie (DGMS) durchgeführt (*n* = 100).

**Ergebnisse:**

Bei der Nutzung der digitalen Technologien in den Veranstaltungen rangieren auf der obersten Stelle Präsentationstools, gefolgt von Lernmanagementsystemen, Videoangeboten sowie digitalen Texten. Die Teilnehmenden geben hinsichtlich der Nutzung unterschiedliche (Konferenz‑)Tools an. Das Erstellen von Lehrvideos bejahen 53 % der Befragten, Abstimmungstools) werden bis > 50 % als unbekannt genannt. Als Herausforderungen werden digitale Infrastruktur der Hochschulen, fehlende didaktische Beratung/Unterstützung sowie rechtliche Fragen (Nutzungsrechte, Datenschutz) angegeben.

**Schlussfolgerung:**

Neue Technologie werden vorwiegend für Wissenserwerb, Wissensvermittlung genutzt, selten zur Aktivierung von Studierenden und zur Gestaltung kollaborativer Lehr- und Lernarrangements sowie Neugestaltung von Lernaufgaben und Lernprozessen (individualisiertes Lernen). Welche der aktuell erprobten digitalen Lehr- und Lernformate zukünftig in der Hochschullehre zunehmend eingesetzt werden, hängt von vielen Faktoren ab, u. a. von digitaler Kompetenz sowie der Bereitschaft der Lehrenden und Lernenden die digitale Lernkultur mitzugestalten.

**Zusatzmaterial online:**

Zusätzliche Informationen sind in der Online-Version dieses Artikels (10.1007/s11553-022-00937-1) enthalten.

## Abstract (en)

**Background:**

The coronavirus disease 2019 (COVID-19) pandemic has driven the digitisation in higher education institutions. At the same time, digital higher education teaching faced the challenge of designing teaching in the context of pre-existing resources, digital and didactic skills, and available technical infrastructure.

**Objective:**

The aim of the survey was to assess the assessment/evaluation of digital teaching, which has largely or completely replaced face-to-face teaching as a result of the COVID-19 pandemic, from the perspective of lecturers in public health, medicine and nursing.

**Materials and methods:**

The cross-sectional survey took place online from June to August 2020 and data were collected via www.soscisurvey. The written survey was conducted among members of the German Society for Public Health (DGPH) and the Digitisation Committee of the Society for Medical Education (GMA) and the Education and Counselling Sections of the German Society for Nursing Science (DGP), and the Teaching Working Group of the German Society for Medical Sociology (DGMS) (*n* = 100).

**Results:**

In terms of the use of digital technologies in the courses, presentation tools ranked first, followed by learning management systems, video content and digital texts. With regard to the use of tools, participants most frequently mentioned various video conference tools). The creation of instructional videos was affirmed by 53% of the respondents; voting tools were mentioned as unknown by over 50%. Digital infrastructure of the universities, lack of didactic advice/support and legal questions (rights of use, data protection) were cited as challenges.

**Conclusion:**

New technology is mainly used for knowledge acquisition, knowledge transfer, rarely for activating students and designing collaborative teaching and learning arrangements as well as redesigning learning tasks and learning processes (individualised learning). Which of the currently tested digital teaching and learning formats will be increasingly used in university teaching in the future depends on many factors, including digital competence and the willingness of teachers and students to help develop the digital learning culture.

## Hintergrund und Fragestellung

Durch die COVID-19(„coronavirus disease 2019“)-Pandemie wurden Krankenhäuser, Schulen und Hochschulen gezwungen, neue digitale Wege zu gehen, um sowohl Versorgung als auch Lehre aufrecht zu erhalten. Der Umstellungsprozess erforderte schnelle Maßnahmen und führte in der Bildung oftmals zu dem viel zitierten Begriff des „emergency remote teachings“ [14]. Diese neuen Herausforderungen stellen aber auch eine Bewährungsprobe und Chancen für Innovationen für die digitale Lehre dar.

Vor der COVID-19-Pandemie gab es bereits gute Entwicklungen des digitalen Lernens und Lehrens [7, 25]. In der Grundlagenforschung bestehen (einige) Evidenzen, die die Vorteile des gezielten Einsatzes der digitalen Lehre herausstellen konnten [9, 21, 30, 31, 35]. So zeigten beispielsweise insbesondere Blended-learning-Formate höhere Effektstärken bei den Learning Outcomes im Vergleich zu der traditionellen Vorlesung [31]. In der Hochschuldidaktik der Studiengänge der Gesundheitsberufe wurde auf Blended-learning-Szenarien gesetzt, aber auch auf den Einsatz von „virtual patients“ [20], Simulationen [4] und spielbasierten Ansätzen [10, 33]. In der medizinischen Ausbildung wurde weitestgehend das „inverted classroom model“ genutzt [3, 34]; in Gesundheitsberufen, u. a. in der Pflege(aus)bildung wurden zunehmend Lehrangebote wie „flipped classroom“ sowie Simulationen eingesetzt [2, 17, 24].

Es gab aber auch relevante Hürden, die die digitale Lehre ausgebremst haben. Mangelnde Erfahrung der Lehrpersonen, Rahmenbedingungen auf verschiedenen gesundheits- und bildungspolitischen Ebenen [12] und mangelnde Transparenz bei der Anrechenbarkeit von digitalem Unterricht [1] sorgten dafür, dass sich die digitale Lehre nur schrittweise weiterentwickeln konnte. Die oben genannten Studienergebnisse kamen nur langsam in der Praxis der Lehre an.

Die COVID-19-Pandemie hat die Digitalisierung an Hochschulen vorangetrieben und zugleich die Frage nach Ausgestaltung der Lehre, technischer Infrastruktur und digitaler Kompetenz aufgeworfen. In diesem Kontext wurden folgende Forschungsfragen formuliert: (1) Welche Lehr- und Lernformen wurden im ersten digitalen Semester (während der COVID-19-Pandemie) genutzt und (2) wie wurde die digitale Lehre der Studiengänge Public Health, Medizin und Pflege eingeschätzt?

## Methodik

Die Querschnitterhebung fand online von Juni bis August 2020 statt und die Daten wurden über www.soscisurvey.de werbefrei ausschließlich zu wissenschaftlichen Zwecken erhoben. Die schriftliche Befragung wurde durch den Fachbereich Lehre der Deutschen Gesellschaft für Public Health (DGPH) und den Ausschuss Digitalisierung der Gesellschaft für medizinische Ausbildung (GMA) konzipiert und unter Mitgliedern mit Beteiligung der Sektionen Bildung und Beratung der Deutschen Gesellschaft für Pflegewissenschaft (DGP) und der Arbeitsgruppe Lehre der Deutschen Gesellschaft für Medizinische Soziologie (DGMS) durchgeführt. Damit konnten Lehrende an Universitäten und Hochschulen aus den Disziplinen Public Health/Gesundheitswissenschaften, Gesundheitspädagogik, Medizin, Pflege und Lehramt (Gesundheit und Pflege) erreicht werden. Geschätzt (laut der Angaben der jeweiligen Fachgesellschaft) ca. 1700 Mitglieder (die an Hochschulen tätig sind) erhielten innerhalb des Newsletters der jeweiligen Gesellschaft Zugang zur Umfrage über einen Weblink. Personalisierte Teilnahmelinks kamen aufgrund datenschutzrechtlicher Bestimmungen im Umgang mit Mitgliedsdaten nicht in Betracht. Neben den Zielen wurde auf der Startseite der Umfrage auf die freiwillige Teilnahme sowie anonymisierte Datenverarbeitung verwiesen und von Teilnehmenden die Einverständniserklärung zum Datenschutz vor der weiteren Bearbeitung eingeholt. Ein umfassendes Datenschutzkonzept stand weiterführend zum Download bereit. Zutreffende Leitlinien zur Sicherung guter wissenschaftlicher Praxis wurden beachtet [5].

Das Ziel der Umfrage war die Beschreibung und Einschätzung der digitalen Lehre aus Sicht der Dozierenden, die die Präsenzlehre infolge der COVID-19-Pandemie im ersten digitalen Semester neu konzipiert haben.

In der standardisierten Erhebung mit überwiegend geschlossenen Fragen wurden Daten zu den Themen Einsatz und Anwendung digitaler Technologien in der Lehre, Tools für digitale Lehre, digitale Lehr-Lern-Konzepte, Erfahrungen und Einstellungen der Lehrenden, Herausforderungen in der digitalen Lehre sowie Evaluationskonzepte und Datenschutz erhoben. Die Umfrage war an den „Monitor Digitale Bildung“ der Bertelsmann Stiftung aus dem Jahr 2017 angelehnt [23]. Der Online-Survey wurde in einem Pretest funktional und sprachlich überprüft und angepasst. Der Link zur Umfrage wurde von 313 Mitgliedern unabhängig davon angeklickt, ob der Fragebogen anschließend wieder geschlossen, nur die Einleitung gelesen oder dieser weiterbearbeitet wurde. Der Nettostichprobenumfang der „convenience sample“ lag bei 100 Teilnehmenden. Die Datenaufbereitung und -auswertung erfolgte mittels SPSS (Version 25); offene Fragen wurden zunächst induktiv kategorisiert (angelehnt an Mayring [22]), aufbereitet und anschließend kodiert.

## Ergebnisse

**Abb. 1:**
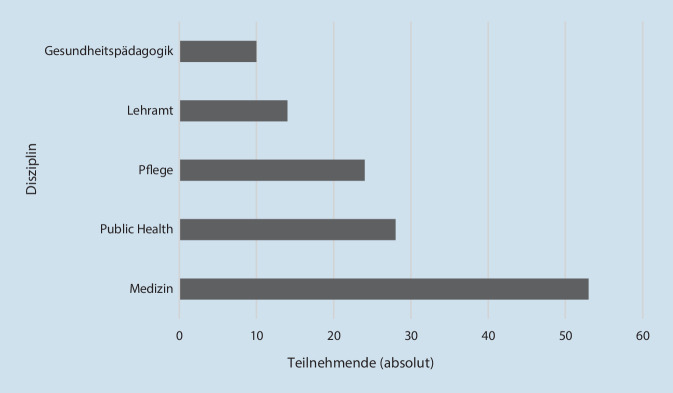
Teilnehmende nach Disziplin (Mehrfachantworten)

An der Umfrage haben Lehrende an Universitäten zu 75 % und 25 % an (Fach)Hochschulen teilgenommen; 60 % haben weiblich, 38 % männlich, 2 % divers angegeben. Die Teilnehmenden sind zu 44 % Mitglieder der GMA, zu 14 % Mitglieder der DGPH, zu 10 % Mitglieder der DGP und bei 32 % ist andere oder keine Mitgliedschaft angegeben. Die Fachdisziplinen, die die Teilnehmenden lehren, umfassen Medizin, Public Health, Pflege und Gesundheitspädagogik (Abb. 1).

Zwar unterscheiden sich die Befragten im Hinblick auf das Alter, die Statusgruppe und Berufsjahre, doch verfügt die Mehrheit über längere Erfahrung in der Hochschullehre (Tab. 1, 2 und 3).

**Tab. 1:** Alter der Befragten

Alter der Teilnehmenden (Jahre)	%
18–29	6
30–39	24
40–49	30
50–59	29
60 oder älter	11

**Tab. 2:** Statusgruppen

Statusgruppe	%
Wissenschaftliche Mitarbeiterinnen und Mitarbeiter	47
Professorinnen und Professoren	32
Lehrbeauftragte	18
Sonstige	3

**Tab. 3:** Erfahrung in der Hochschullehre

Berufsjahre der Teilnehmenden	%
Weniger als 1	8
1–5	20
6–10	17
11–20	29
21 und länger	26

## Technologien in der Hochschullehre

Bei der Nutzung der digitalen Technologien in den Veranstaltungen rangieren auf der obersten Stelle Präsentationstools, gefolgt von Lernmanagementsystemen, Videoangeboten sowie digitalen Texten (Tab. 4).

**Tab. 4:** Nutzung der Technologien in Veranstaltungen (Mehrfachantworten)

Technologie	Nutzung (%)
Präsentationstools	92
Lernmanagementsysteme (OLAT etc.)	76
Videoangebote (YouTube etc.)	67
Digitale Texte	63
Elektronische Tests	55
Office, Programme	47
Datenbanken	30
Response-Systeme (Mentimeter etc.)	29
Cloud-Dienste	27
Foren, Communities, Blogs	26
Software (Statistik etc.)	23
Digitale Spiele/Simulationen	19
E‑Portfolios	19
Wikipedia	16
Lern-Apps	9
Chat-Dienste	6
MOOC („massive open online courses“)	5
Soziale Netzwerke	2

Hinsichtlich der Nutzung von (Konferenz)Tools geben die Teilnehmenden am häufigsten Zoom (Zoom, San Jose, CA, USA) (52 %), an. Zugleich wird die große Spannbreite von Big Blue Button (BigBlueButton Inc., Ottawa, Kanada) (22 %), Adobe Connect (Adobe, San Jose, CA, USA) (24 %), MS Teams (MS Teams, Redmond, WA, USA) (25 %), Cisco Webex (Cisco, Milpitas, CA, USA) (22 %) ersichtlich.

Für Abstimmungen in den Seminaren werden v. a. in den hochschulinternen Tools integrierte Abstimmungstools zu 37 % genannt. Als unbekannt werden folgende Onlineformate angegeben: Slido (Slido, Bratislava, Slovakei) (zu 67 %), Tweedback (Tweedback GmbH, Rostock, Deutschland) (65 %), OnlineTed (Online TED® Erlangen, Deutschland) (59 %), auch Kahoot! (Kahoot!, Oslo, Norwegen), Pingo (Pingo, Paderborn, Deutschland) und Mentimeter (Mentimeter, Stockholm, Schweden) bleiben jeweils mit > 50 % unbekannt.

**Abb. 2:**
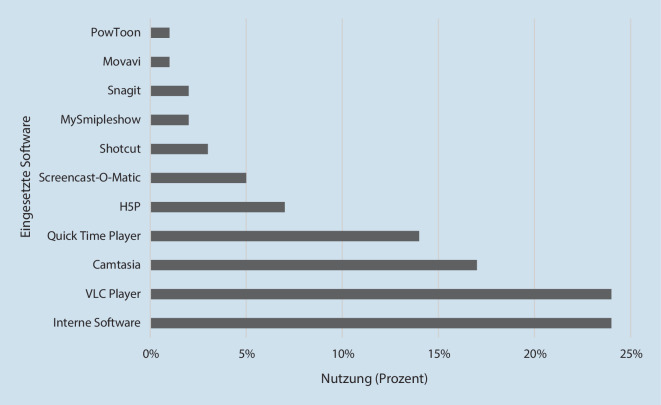
Eingesetzte Software zur Erstellung von Lernvideos

Das Erstellen von Lehrvideos bejahen 53 % der Befragten, wohingegen 47 % der Teilnehmenden keine Videos erstellen. In dem Einsatz von Videosoftware werden am häufigsten interne Software genannt (Abb. 2).

## Lehr- und Lernarrangements: Anwendung und Einschätzung

**Abb. 3:**
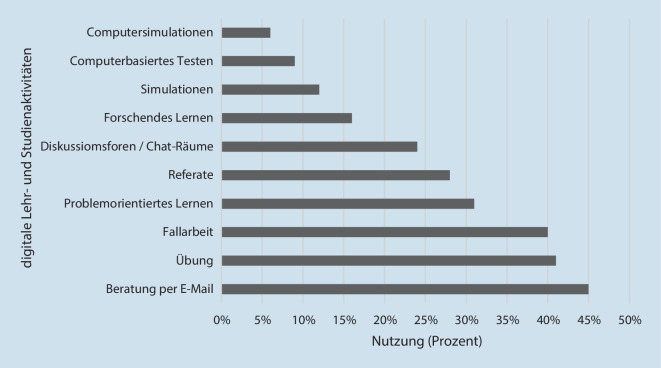
Digitale Lehr- und Studienaktivitäten

Der Einsatz digitaler Technologien zeigt ein vielfältiges Bild bezogen auf Lern- und Studienaktivitäten in der Onlinelehre (Abb. 3). Dabei wurden folgende Formate in digitaler Form am häufigsten eingesetzt: Beratung per E‑Mail, Übungen sowie Fallarbeit und problemorientiertes Lernen, während die genuin digitalen Formen wie Computersimulationen sowie computerbasiertes Testen nur sporadisch angegeben wurden.

Die Einschätzung der Outcomes im Kontext der eingesetzten digitalen Lehrkonzepte zeigt gewisse Tendenzen auf: Präsentationstools, Lernvideos sowie PDF-Dokumente und eBooks wurden primär für die Wissensvermittlung eingesetzt. Bei Erstellung von Projekten mit digitalen Medien werden – nach Ansicht der Teilnehmenden – soziale und digitale Kompetenzen sowie Selbstorganisation, Selbstständigkeit und Anwendung von Wissen erwartet. Der Einsatz von Selbstlernprogrammen lässt nach Einschätzung der Befragten vor allem digitale Kompetenz sowie Selbstständigkeit erzielen, weniger Wissensvermittlung und Anwendung von Wissen (Tab. 5).

**Tab. 5:** Einsatz von digitalen Lehrkonzepten im Kontext von Outcomes

	Wissensvermittlung (%)	Anwendung von Wissen (%)	Sozialkompetenz (%)	Selbstständigkeit (%)	Selbstorganisation (%)	Digitale Kompetenz (%)
Lernvideos/Präsentationstools/Whiteboards	80	36	7	12	19	32
PDF-Dokumente/eBooks	68	17	2	28	33	15
Response-Systeme	14	22	16	14	5	20
Lernmanagementsystem	46	36	18	59	64	52
Selbstlernprogramme (Lern-Apps, Simulation)	16	28	10	36	29	31
„Inverted classroom“	29	48	33	62	62	40
Erstellung von Projekten mit digitalen Medien	12	54	63	58	59	60

Des Weiteren wurden als bewährte Lehr- und Lernangebote folgende Formate genannt:

- Vortrag mit Lernvideos/Präsentationstools/Whiteboard 76 %,
- Lernmanagementsysteme (Moodle [Moodle, West Perth, Australien], ILIAS [The open source Learning Management System, Köln, Deutschland]) 67 %,
- PDF-Dokumente/eBooks 59 %.

Für die Zukunft werden insbesondere Lernvideos sowie vertonte PowerPoint-Präsentation und Videokonferenzen als versprechend eingeschätzt.

Inwiefern digitale Lehr- und Lernformate den Kompetenzerwerb der Studierenden fördern, wurde unterschiedlich bewertet: 15 % der Befragten sind der Ansicht, dass digitale Lehre Kompetenzerwerb verbessert, 16 % stimmen dagegen, 48 % stimmen eher zu, 21 % stimmen eher dagegen. Als die häufigsten Sozialformen in den Onlineseminaren wurde die Einzelarbeit (58 %), gefolgt von Arbeit im Plenum (57 %) angegeben. Demgegenüber werden Partnerarbeit mit 25 % und Gruppenarbeit mit 33 % als häufig praktizierende Sozialformen angeführt.

Die Befragten geben bei ihrer Einschätzung an, dass digitale Lehre weder Dozierende entlastet (57 %), Abbruchquoten im Studium verringert (63 %), noch sozial benachteiligen Lernenden den Zugang verbessert (61 % können dem Item nicht zustimmen). Außerdem werden die Angebote als aufwändig betrachtet (80 % Zustimmung). Lediglich bei körperlich beeinträchtigen Lernenden wird digitale Lehre als positiv eingeschätzt (50 % Zustimmung), d. h. erleichtert den Zugang zu Hochschullehre. Die Einschätzung der technischen Ausstattung zum digitalen Lernen an der jeweiligen Hochschule wurde zu 33 % als gut bewertet, als völlig unzureichend und unzureichend (20 %); knapp die Hälfte der Einschätzung liegt im Mittelfeld („eher gut“ und „eher unzureichend“) mit 47 %.

Die Zufriedenheit der Lehrenden ist insbesondere im Hinblick auf die Umsetzung digital gestützter Lehre in Seminaren sowie der Kommunikation mit den Studierenden gegeben. Geteilter Meinung sind die Teilnehmenden im Hinblick auf den kollegialen Austausch sowie die Durchführung der Lehre aus dem Homeoffice.

## Herausforderungen

Als Herausforderungen der digitalen Lehre werden insbesondere rechtliche Fragen (Nutzungsrechte, Datenschutz) genannt. Dabei variieren die Ansichten hinsichtlich der Beurteilung des Datenschutzes zwischen kritisch (51 %) und unkritisch (42 %); 7 % haben die Frage nicht beantwortet. Die Ergebnisse bezüglich der Speicherung der Onlineseminare bejahten 8 % der Teilnehmenden, 59 % berichten, die Onlineseminare werden nicht gespeichert, 24 % beantwortet die Frage mit „teils-teils“ und 9 % beantwortet die Frage mit „ich weiß es nicht“.

**Abb. 4:**
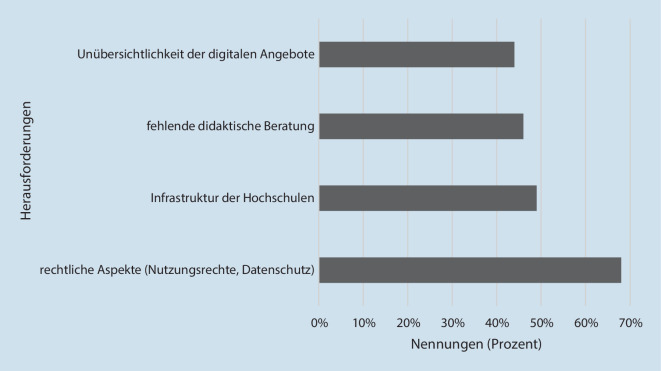
Herausforderungen digitaler Lehre (Mehrfachantworten)

Des Weiteren wird die Unübersichtlichkeit der digitalen Angebote moniert und auch die Unterstützung seitens der Hochschulen bemängelt (Abb. 4).

## Diskussion

Die vorliegenden Ergebnisse zeigten Unterschiede bei der Nutzung von Technologien in Veranstaltungen gegenüber der Befragung für den Monitor Digitale Bildung aus dem Jahr 2017 [29]. In beiden Erhebungen wurde die Verwendung von Präsentationstools in Lehrveranstaltungen von 92 % der Teilnehmenden angegeben.

Ein zunehmender Einsatz wurde in dieser Umfrage u. a. bei elektronischen Tests (55 % gegenüber 28 % nach Schmid [29]), Lernmanagementsystemen (76 % gegenüber 51 %) und Cloud-Diensten (27 % gegenüber 5 %) aufgezeigt. Demgegenüber wurden Officeprogramme (47 % gegenüber 64 % nach Schmid [29]) und Software (23 % gegenüber 36 %) im COVID-19-Semester seltener eingesetzt. Der Einsatz von Wikipedia (16 % gegenüber 27 %) war gleichermaßen rückläufig.

Die Umstellung der Präsenzlehre auf digitale Lehre im ersten Semester der Coronapandemie offenbarte eine große Vielfalt an digitalen Tools und deren Einsatz. Die vorliegende Studie lässt die positive Tendenz zur Einschätzung digitaler Lehre im Studium der Gesundheitsberufe erkennen. Zugleich werden Herausforderungen bei der Vielfalt der digitalen Tools und der adäquaten Auswahl beim Einsatz der digitalen Technologien unverkennbar, die bereits vor dem ersten digitalen Semester ohne Präsenz in zahlreichen Publikationen und Studien thematisiert worden sind [11]. Die Erkenntnisse verdeutlichen den Bedarf an professioneller Betreuung der digitalen Infrastruktur – insbesondere hinsichtlich didaktischer aber auch technischer Unterstützung an Hochschulen.

Der Einsatz neuer Medien in unserer Untersuchung hat nach dem SAMR-Modell [26] vorwiegend die Funktion der Substitution (Ersatz der Lernwerkzeuge, ohne funktionelle Änderung) und Augmentation (Steigerung, d. h. digitale Medien werden als Ersatz für Lernwerkzeuge betrachtet, die funktionelle Verbesserung zu erzielen suchen), weniger Modifikation (Neugestaltung von Lernaufgaben) und Redefinition (Möglichkeit digitale Medien zur Realisierung von Lernaufgaben). So werden beispielsweise Präsentationstools und digitale Texte bzw. Lernvideos in den Veranstaltungen eingesetzt, die eher als Ersatz der Präsenzlehre ggf. Augmentation gelten. Simulationen, E‑Portfolio oder Lern-Apps finden dagegen wenig Anwendung. Im Kontext der Sozialformen überwiegte in unserer Studie die Einzelarbeit und Arbeit im Plenum im Vergleich zu kooperativen Lernformen in Gruppen (u. a. Projektarbeiten). Insofern wird die Neugestaltung von Lernaufgaben und Lernprozessen (insbesondere Formen des individualisierten Lernens) selten praktiziert und damit bleiben die Potenziale neuer Technologien nicht ausgeschöpft. Die Erkenntnisse zeigen ähnliche Tendenzen wie die ersten Studien an Schulen im deutschsprachigen Raum während der Pandemiezeit [6, 16]. An den Hochschulen (im Gesundheitsbereich) wurden ebenfalls viele unterschiedliche digitale Lernkonzepte umgesetzt; der Schwerpunkt lag auf digitalen Prüfungen, Kommunikationskursen und Kursen zur Vermittlung von Fertigkeiten [32].

Die einseitige Nutzung neuer Technologien wird auch im Hinblick auf Videoeinsatz und Kompetenzerwerb in der Forschung dokumentiert: Videos werden vorwiegend zur Wissensvermittlung bzw. Wissensaneignung genutzt; Einsatzzwecke zur Kommunikation und Kooperation bzw. zur Reflexion von Lerninhalten sind dagegen rar [28]. Frühere Untersuchungen der Hochschullehre haben Potenziale digitaler Technologien u. a. hinsichtlich Aktivierung von Studierenden und Selbstbestimmung, Gestaltung kollaborativer Lehr- und Lernarrangements und Reflexionsprozesse aufgezeigt [27].

Der Erfolg in Onlinekursen hängt vorwiegend von der Betreuung durch Dozierende, eine abwechslungsreiche Konzeption der Inhalte und dem Wechsel zwischen sozialen Lernformen mit der Möglichkeit zum Austausch ab [36]. Schließlich wird ein adäquater Einsatz digitaler Technologien und deren Potenziale vermisst und die Unterstützung der Lehrpersonen im Kontext didaktisch-methodischer Maßnahmen nahegelegt. Des Weiteren ist evidenzbasierte Forschung der digitalen Lehre gefordert, die die Wirksamkeit im Hinblick auf Kompetenzerwerb untersucht.

Eine Unstimmigkeit bezog sich auf den Datenschutz, der teils kritisch teils unkritisch bewertet wurde. Inwiefern die Kenntnisse fehlten oder aufgrund der Alternativlosigkeit in der Lehre der Datenschutz unterschiedlich eingeschätzt wird, lässt sich aus den Daten nicht ermitteln. Gerade zu Beginn der Pandemie sind Unsicherheiten hinsichtlich des Datenschutzes anzunehmen, zumal keine einheitlichen Vorgaben innerhalb der Hochschulen existierten und Lehrende auf schnell verfügbare Lösungen vertrauten. Ob Kenntnisse im Aufbau und der Datenverarbeitung der Programme sowie Verarbeitung personenbezogener Daten vorhanden waren, ob juristische Konsequenzen bekannt waren, welche Rolle die Hochschulen und einzelne Dozierende übernehmen (sollten), lässt viele Erklärungsmöglichkeiten zu, die in weiteren Studien untersucht werden können.

Zusammenfassend kann festgehalten werden, dass infolge der Ad-hoc-Digitalisierung der Lehre das Studium der Public Health, Medizin und Pflege digital konzipiert, synchrone und asynchrone Lernformate eingesetzt wurden sowie prinzipielle Zufriedenheit aus der Perspektive der Lehrenden ersichtlich wird. Tendenziell wurden jedoch die Lehr- und Lernform der Präsenzvorstellung auf die digitale Lehre transformiert, sodass die Formen des Vortragens und Lesens (z. B. PDF-Dateien) dominierten. Hingegen wurden kooperative Arbeitsformen lediglich sporadisch genutzt. Insofern sind zukünftig Potenziale der digitalen Technologien in der Hochschuldidaktik konzeptionell sowie deren Wirksamkeit empirisch zu forcieren.

In welchem Ausmaß diesem Anspruch im zweiten, pandemiebedingten Onlinesemester noch stärker nachgekommen werden konnte und welche Optionen für den studentischen Austausch außerhalb der Veranstaltungen geschaffen wurden, kann Gegenstand weiterer Forschungsarbeiten sein.

## Limitation

Die Ergebnisse zeigen Limitationen im Hinblick auf die kleine Zahl der Teilnehmenden (auch im Hinblick auf Convenience-sample-Ansatz), sodass repräsentative Erkenntnisse nicht möglich sind. Die Stärke der Studie ist in der Beteiligung der DGPH, der medizinischen Ausbildung (GMA) und Pflegewissenschaft (DGP) sowie der DGMS zu sehen, die sich in den jeweiligen Fachbereichen, Arbeitsgruppen und Sektion der Lehre/Bildung engagieren. Alle Mitglieder der angefragten Gesellschaften wurden angefragt, sich an der Umfrage zu beteiligen. Die Einschätzung der digitalen Lehre erfolgte aus der Sicht der Lehrenden. Die Perspektive der Studierenden würde die Ergebnisse (insbesondere bezogen auf Kompetenzentwicklung) vervollständigen und einen Vergleich ermöglichen.

## Ausblick

Inwiefern digitale Lehre langfristig erfolgreich sein wird, hängt zum einen von Rahmenbedingungen der Hochschulen – sowohl Infrastruktur, Support als auch Unterstützungsangebote – und zum anderen von digitaler Kompetenz der Lehrenden und Lernenden ab. Unter digitaler Kompetenz wird sowohl das Anwenden der Technologien, Anleiten, Beraten und Unterstützen von mediengestützten Lehr- und Lernsettings als auch das Präsentieren und Produzieren sowie Analysieren, Reflektieren, Kommunizieren und Kooperieren subsummiert [8, 15]. Hierbei fokussieren wir in der Hochschuldidaktik digitale Kompetenz der Lehrenden. Zugleich ist die häufig angenommene digitale Kompetenz der Studierenden, die als „digital natives“ bezeichnet werden, als „myths of the digitale native“ zu problematisieren [18, 19]. Folglich ist das Anwenden, Analysieren und Reflektieren im Kontext der von Outcomes, die auf dem Arbeitsmarkt gefordert werden, multiperspektivisch zu betrachten: aus Sicht der Lehrenden und Lernenden, vor dem Hintergrund digitaler Technologien sowie deren Potenziale.

In diesem Kontext bleibt die Frage nach digitaler Lernkultur in der Hochschuldidaktik der Public Health, Medizin und Pflege, die selbstbestimmtes, individualisiertes und partizipatives Lernen ermöglichen kann. Zukunftsweisend ist strukturiertes Feedback, kritisches Denken und (Selbst‑)Reflexion, die in den selbstbestimmten Lernprozessen vorausgesetzt werden – Aspekte, die Priorität in der COVID-19-Zeit erfahren (haben).

Der Fokus in der Hochschullehre ist nicht auf „technisch Machbares“ zu legen, sondern auf „didaktisch Wünschenswertes“, denn sobald Digitalisierungsbemühungen „den Technologien das Feld überlassen“, „werden digitale Szenarien kaum mehr sein als die eines „Framework“ für Inhalte“ [13]. Insofern dürfte digitale Lernkultur in den Mittelpunkt der Hochschuldidaktik rücken: Gestaltung der Lehr- und Lernarrangements, digitale Kompetenz sowie bedarfs- und bedürfnisorientierte Unterstützungsangebote an Hochschulen. Hierzu bedarf es empirischer Erkenntnisse zur Wirksamkeit der Lehrkonzepte und Methodik, zum Bedarf der Lehrenden hinsichtlich der digitalen Lehr- und Lernformate sowie zum Erwerb digitaler Kompetenz der Lehrenden und Lernenden.

## Fazit für die Praxis

- Digitale Hochschullehre in Public Health, Medizin, Pflege erfordert eine didaktisch-methodische Entwicklung: Projektarbeit mit digitalen Lehr- und Lernformen zum Fördern des selbstorganisierten Lernens und digitaler Kompetenz, kollaborative Lehr- und Lernarrangements zum Fördern kommunikativer und sozialer Kompetenz, Lern-Apps zum selbstbestimmten sowie zeit- und ortsunabhängigen Lernen.
- Es werden passgenaue Weiterbildungsangebote benötigt, deren Konzeption auf dem Bedarf in der Lehre und den Bedürfnissen der Dozierenden basiert.
- Die Verankerung der Nutzungsrechte und des Datenschutzes in der Hochschuldidaktik kann zur digitalen Transparenz und Souverenität der Dozierenden beitragen.
- Im Sinne „open education ressource“ eröffnet internationale und hochschulübergreifende Vernetzung zahlreiche Lehr- und Lernpotenziale.
- Adäquate Maßnahmen und Strukturen an Hochschulen können die Potenziale der digitalen Technologien optimal nutzen und weiterentwickeln.
- Für digitale Hochschuldidaktik sind sowohl digitale Kompetenz der Lehrenden und Lernenden sowie digitale Lernkultur zentral und damit zukünftig zu fokussieren.

## Supplementary Information

Übersicht von benannten Produkten
