# Supplementary material for: Digital higher education in the first COVID-19 semester. A survey of teachers in public health, medicine and nursing
Source: Pravent Gesundh. 2022 Mar 20;18(1):22–9. [Article in German] doi: 10.1007/s11553-022-00937-1 (PMC8934585; doi:10.1007/s11553-022-00937-1)
Supplement: Supplementary file 1 — Übersicht von benannten Produkten [file 11553_2022_937_MOESM1_ESM.docx]

**Übersicht von benannten Produkten**

**Programm, Firma, Ort, Bundesstaat, Land**

AdobeConnect, Adobe, San Jose, CA, USA

Big Blue Button, BigBlueButton Inc., Ottawa, Kanada

Camtasia, Techsmith, Okemos, MI, USA

HSP, HSP, Hamburg, Deutschland

Ilias, The open source Learning Management System, Köln, Deutschland

Kahoot!, Kahoot!, Oslo, Norwegen

Mentimeter, Mentimeter, Stockholm, Schweden

Microsoft Office, Microsoft, Redmond & Washington, USA

Microsoft Teams, Microsoft, Redmond USA

Moodle, Moodle, West Perth, WA, Australien

Movavi, Movavi, Wildwood, MO, USA

Mysimpleshow, Simpleshow, Berlin, Deutschland

Online Ted, Online TED^®^, Erlangen, Deutschland

Open Olat, OLAT, Zürich, Schweiz

Pdf, Adobe, San Jose, USA

Pingo, Pingo, Paderborn, Deutschland

Powtoon, Powtoon, London, UK

Quicktime Player, Apple, Cupertino, CA, USA

Shotcut, Meltytech, Oceanside, CA, USA

Slido, Bratislava, Slovakei

Snagit, Techsmith, Okemos, MI, USA

SPSS, IBM, Armonk, N.Y., USA

Screencast-O-Matic, Big Nerd Software, Seattle, WA, USA

Tweedback, Tweedback GmbH, Rostock, Deutschland

VLC Player, Videolan, Châtenay-Malabry, Frankreich

Webex by Cisco, Milpitas, CA, USA

YouTube, YouTube, LLC, San Bruno, USA

Zoom, Zoom, San Jose, CA, USA
